# A retrospective study on epidemiology and management of canine cystine uroliths in one part of Norway from 2015 to 2020

**DOI:** 10.1186/s13028-023-00711-z

**Published:** 2023-11-14

**Authors:** Terese Vatne Naeverdal, Janne Eidissen Midtgård, Ann-Katrin Llarena, Martine Lund Ziener

**Affiliations:** 1 EMPET AS, Strømsveien 163, 1466 Strømmen, Norway; 2 Food Safety Unit, Department of Paraclinical Sciences, Faculty of Veterinary Medicine, Norwegian University of Life Sciences, Ås, Norway; 3 Fredrikstad Animal Hospital, Wilbergjordet 2, 1605 Fredrikstad, Norway

**Keywords:** Age, Breed, COLA, Occurrence

## Abstract

**Background:**

Urinary tract problems are a common complaint in small animal medicine and urolithiasis is considered to be an important cause of urinary tract disease in dogs. In this study the main aim was to investigate whether the occurrence of cystine urolithiasis increased during a five-year period. A second aim was to evaluate possible risk-factors as breed, age and gender. This study also evaluated how urine specific gravity, pH and level of cystine in urine responded to preventive strategies. Medical records of dogs with urolithiasis presented at nine Norwegian animal clinics and one animal hospital between 2015 and 2020 were retrospectively reviewed.

**Results:**

The incidence of cystine uroliths increased significantly during the five study years (R^2^ = 0.72, P = 0.0199). Dogs with cystine uroliths were significantly younger (5.0 years (n = 84, 95% CI [4.4–5.6])) when they were diagnosed with cystine uroliths compared to dogs with other types of uroliths (8.1 years (n = 255, 95% CI[7.8-8.5]) P < < 0.0001). Cystine levels in urine were increased in 93% of the dogs with cystine urolithiasis. Cystinuria decreased significantly after neutering (P < 0.0001). Breeds most commonly affected with cystine urolithiasis in this study were Staffordshire bull terrier, Danish Swedish farmdog and Chihuahua.

**Conclusions:**

The results from this study supports a suggested genetic basis for cystine urolithiasis as described in previous studies. Neutering is considered an important part of preventing reoccurrence since cystine values decreased significantly after neutering.

## Background

Cystinuria is caused by an inherited proximal renal tubular defect in which reabsorption of the amino acid cystine, and to a variable extent lysin, arginine and ornithine (COLA), from the glomerular filtrate fails [1]. The solubility of cystine in neutral and acidic urine is low, and cystinuria thereby increases the risk of cystine urolithiasis [1].

The transport defect of cystine appears to be a genetically heterogenous disease. Four types of canine cystinuria have been categorized according to genetic findings; Type I-A, Type II- A, Type II-B and Type III. Different mutations in the gene *SLC3A1* cause Type I-A and Type II-A cystinuria, with respectively autosomal recessive and autosomal dominant inheritance. Type II-B cystinuria is caused by mutations in the gene *SLC7A9* and occur in a pattern being consistent with an autosomal dominant inheritance. Type III cystinuria is limited to male dogs and has an undetermined mode of inheritance [2].

Previous studies have showed an increased occurrence of cystine uroliths in breeds such as Staffordshire bull terrier, English bulldog, Dachshund and Chihuahua among others, depending on which country the study is from. A genetic basis for cystinuria has been suggested [3].

The treatment of clinical cystine urolithiasis involves medical dissolution with tiopronin, removal of stones by voiding urohydropropulsion, basket retrieval, lithotripsy or surgery [4]. Preventing formation of new stones is of great importance due to the relatively high recurrence rate [4]. Reducing urine specific gravity to < 1.020, increasing the urine pH > 7.5, feeding a low-protein diet, neutering in cases of Type III cystinuria and medicating with tiopronin has been suggested to reduce the risk of recurring cystine uroliths [4]. However, because of the heterogeneity of cystine urolithiasis, each case must be treated individually [1]. This makes treatment and prevention of cystine uroliths challenging for both general practitioners and clients.

Here we present a hospital-based retrospective study of the epidemiology and management of cystine urolithiasis from two regions of Norway between 2015 and 2020. We compared occurrence of cystine uroliths to other type of uroliths. We then investigated whether the incidence of cystine urolithiasis increased during the study period and evaluated the suspected risk factors such as breed, age and sex in relation to occurrence of cystine urolithiasis. A second aim was to investigate if and how urine specific gravity, pH and cystine, ornithine, lysine, and arginine (COLA) values responded to common treatment and prevention strategies and neutering, respectively.

## Methods

### Cases and materials

Medical records of canine patients with urolithiasis confirmed by radiographs or ultrasound presented at nine Norwegian small animal clinics and one animal hospital between 2015 and 2020 were retrospectively searched and reviewed. The patients were grouped according to type of urolith; magnesium ammonium phosphate, calcium oxalate, cystine, urate, silica, mixed or unknown. Metadata collected included gender, breed and age at first diagnosis, neutering status and if the dog was treated surgically or not.

The medical records of dogs with laboratory confirmed cystine urolithiasis were reviewed more extensively. Type of diet before and after diagnosis was obtained, either from the medical records or by contacting the owners by e-mail. Details on treatment for cystine urolith cases were retrospectively reviewed and classified as; no treatment, surgery or voiding urohydropropulsion. Prophylactic measures such as if and when the patients had been neutered, surgically or with deslorelinacetat implant or treated with tiopronin was registered.

### Urine analysis

Urine analysis was generally done in-house unless otherwise mentioned, and usually included a dipstick analysis, measurement of specific gravity by refractometer and microscopic evaluation of urine sediment either by manual microscopy or using SediVue Dx (IDEXX). Characterization of the urolith stones was predominately done by optical crystallography and infrared spectroscopy by IDEXX reference laboratory, Minnesota Urolith Center or LABOKLIN laboratories. COLA analysis was performed by liquid chromatography mass spectrometry by the IDEXX or LABOKLIN laboratories. We defined patients as cystinuric according to the proposed reference intervals by LABOKLIN and IDEXX: cystine > 179 umol/g creatinine (1–5 years) and > 225 umol/g creatinine (> 5years) and if the sum of all four amino acids > 700 umol/g creatinine. Two patients were excluded from this group of COLA analysis. One of the patients had inexplicable deviating results and the other one had cystinuria because of Fanconi´s syndrome.

Data on urine analysis on the day of the diagnosis of cystine uroliths and on follow up visits were obtained from the medical records, including time between intervention and urine analysis when relevant.

For breeds with a high incidence of cystine urolithiasis, i.e., Staffordshire bullterrier, Chihuahua and Danish Swedish farm dogs, relative incidences of cystine urolithiasis were calculated relative to number of individuals of that breed visiting the hospitals for other reasons than cystine urolithiasis.

### Data analysis

All descriptive statistics and cross-tabulations were performed in Stata/SE 15.1 (StataCorp LLC, TX, USA) or Microsoft® Excel® 2008 Version 12.3.6 (130,206). To investigate if cystine uroliths in canines had become more frequent during the study years, two linear regression curves were made using cases diagnosed with cystine uroliths as dependent variable (both as absolute and relative numbers) and number of years passed from 2010 (year nil) as independent variable (n = 6). The distribution of age at urolithiasis diagnosis was controlled for normality using the Shapiro-Wilk test [5], and the difference in mean age at time of diagnosis between dogs with cystine uroliths (n = 84) and other types of uroliths (n = 255, one group and independently; Calcium oxalate, not defined, mixed, silica, magnesium ammonium phosphate and urate) was assessed using the independent t-test. Pearson Chi-square or Fisher’s exact test was used to assess the relationship between neuter-status and urolith type for cystine and calcium oxalate uroliths. P-values below 0.05 were regarded as significant. Microsoft® Excel® 2008 Version 12.3.6 (130,206) was used to calculate 95% confidence interval for cystine, ornithine, lysine, and arginine in urine and to evaluate if there was a significant reduction of values after neutering.

## Results

### Occurrence of uroliths over time, age, and gender

**Fig. 1:**
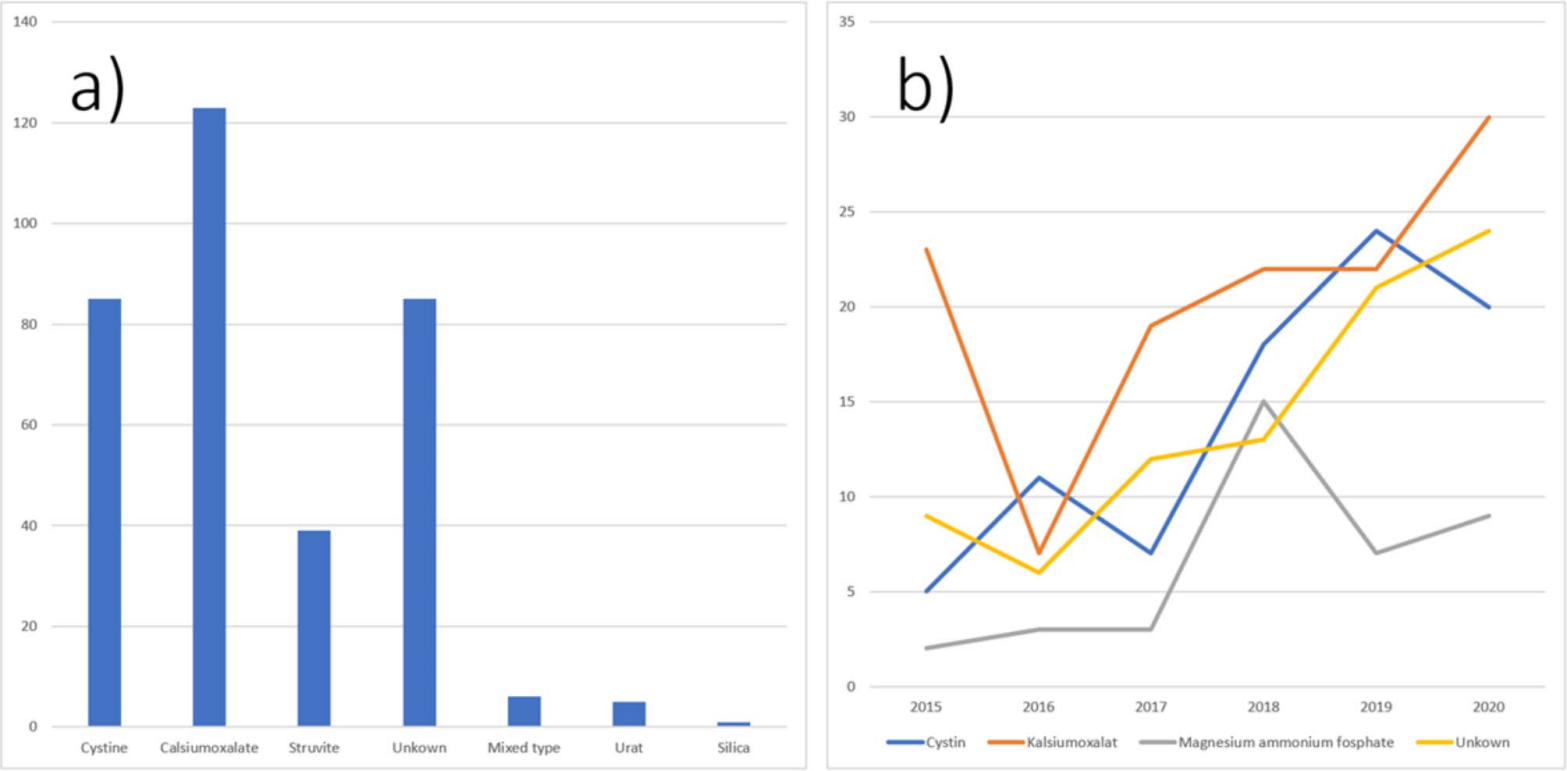
(a) Cumulative number of dogs (y-axis) diagnosed with different urolith types (x-axis) between 2015–2020, and (b) Urolith type per year (2015–2020)

**Fig. 2:**
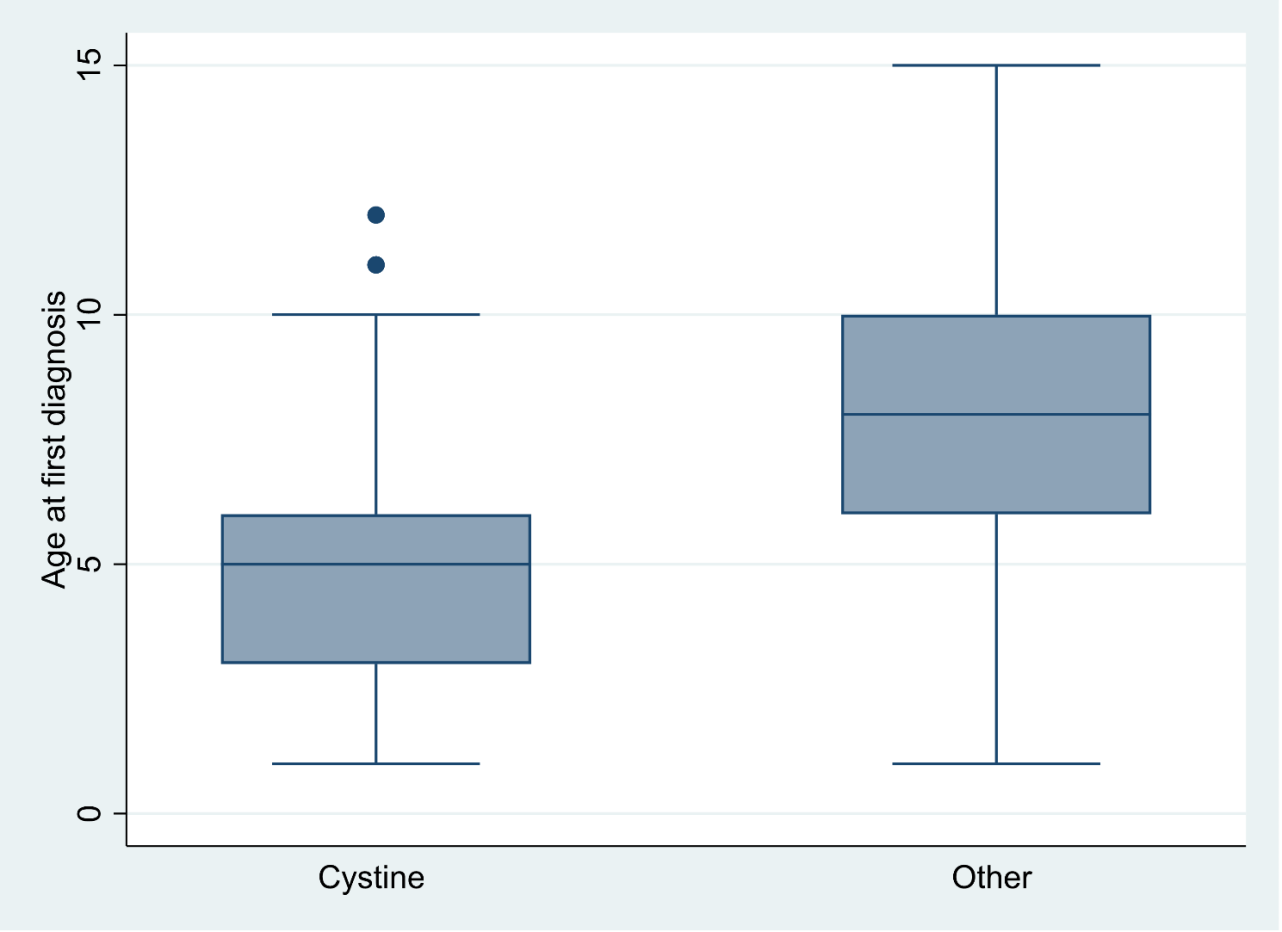
Box-plot of the age at first diagnosis of urine urolithiasis according to type of urolith: cystine vs. calcium oxalate. Lower and upper box boundaries 25th and 75th percentiles, respectively, line inside box median, lower and upper error lines 10th and 90th percentiles, respectively, filled circles data falling outside 10th and 90th percentiles (two male dogs diagnosed with cystine urolithiasis at age 11 and 12)

The total number of patients diagnosed with uroliths at the ten clinics in Norway from 2015 to 2020 were 345. The most common type was calcium containing uroliths (35.6%, n = 123/345), while one quarter of the stones were either cystine (24.6%, n = 85/345) or of unknown composition (24.9%, n = 86/345) (Table 1). The yearly incidence of uroliths, except for magnesium ammonium phosphate, increased between 2015 and 2020 (Fig. 1), and the incidence of cystine uroliths increased significantly during the five study years (linear regression, R^2^ = 0.72, P = 0.0199). Dogs were significantly younger (5.0 years (n = 84, 95% CI [4.4–5.6])) when they were diagnosed with cystine uroliths (Fig. 2) compared to dogs diagnosed with other types of uroliths (8.1 years (n = 255, 95% CI [7.8–8.5]) P < < 0.0001). The dogs diagnosed with cystine urolithiasis were of 34 different breeds (Table 2), of which Staffordshire bull terrier (n = 15), Chihuahuas (n = 10), and Danish Swedish farmdogs (n = 5) had the highest occurrence. Of all the Staffordshire bull terriers that visited the clinics during the five study years, 0.93% (15/1596 Staffordshire bull terriers) were diagnosed with cystine uroliths, while the corresponding figure for Danish Swedish farmdogs (5/570) and Chihuahuas (11/1434) were 0.87% and 0.76%, respectively. In our material, all dogs diagnosed with cystine uroliths were intact male dogs, and male dogs were also overrepresented among dogs with calcium uroliths.

**Table 1:** The most common type of urolith in patients diagnosed with urolithiasis from 2015–2020

Type of urolith	Intact male	Castrated male	Intact female	Castrated female	Total
Cystine	85	0	0	0	85
Calcium oxalate	80	15	25	3	123
Magnesium-ammonium-phosphate	11	3	23	2	39
Unknown	46	11	26	3	86

**Table 2:** Complete list of breeds with cystine uroliths. Breeds listed in alphabetical order

Breed	Number	Breed	Number
Akita	2	Jack Russell terrier	1
Australian sheperd	1	Kromforhrländer	1
Basenji	1	Miniature pincher	1
Bichon frisé	2	Mixed breed	6
Bichon havanais	2	Norbottenspitz	1
Border collie	1	Papillon	1
Cairn terrier	1	Pomeranian	1
Chihuahua	11	Pug	2
Dachshund	3	Rottweiler	2
Dachshund miniature	2	Shetland sheepdog	4
Danish Swedish farmdog	5	Siberian husky	1
Dorset old tyme bulldog	1	Spanish waterdog	2
English bulldog	2	Staffordshire bullterrier	15
English toy terrier	1	Tibetan spaniel	2
Eurasier	1	Welsh corgi	2
Finnish lapphund	1	West highland white terrier	1
French bulldog	2	Whippet	1
Italian greyhound	2		

**Fig. 3:**
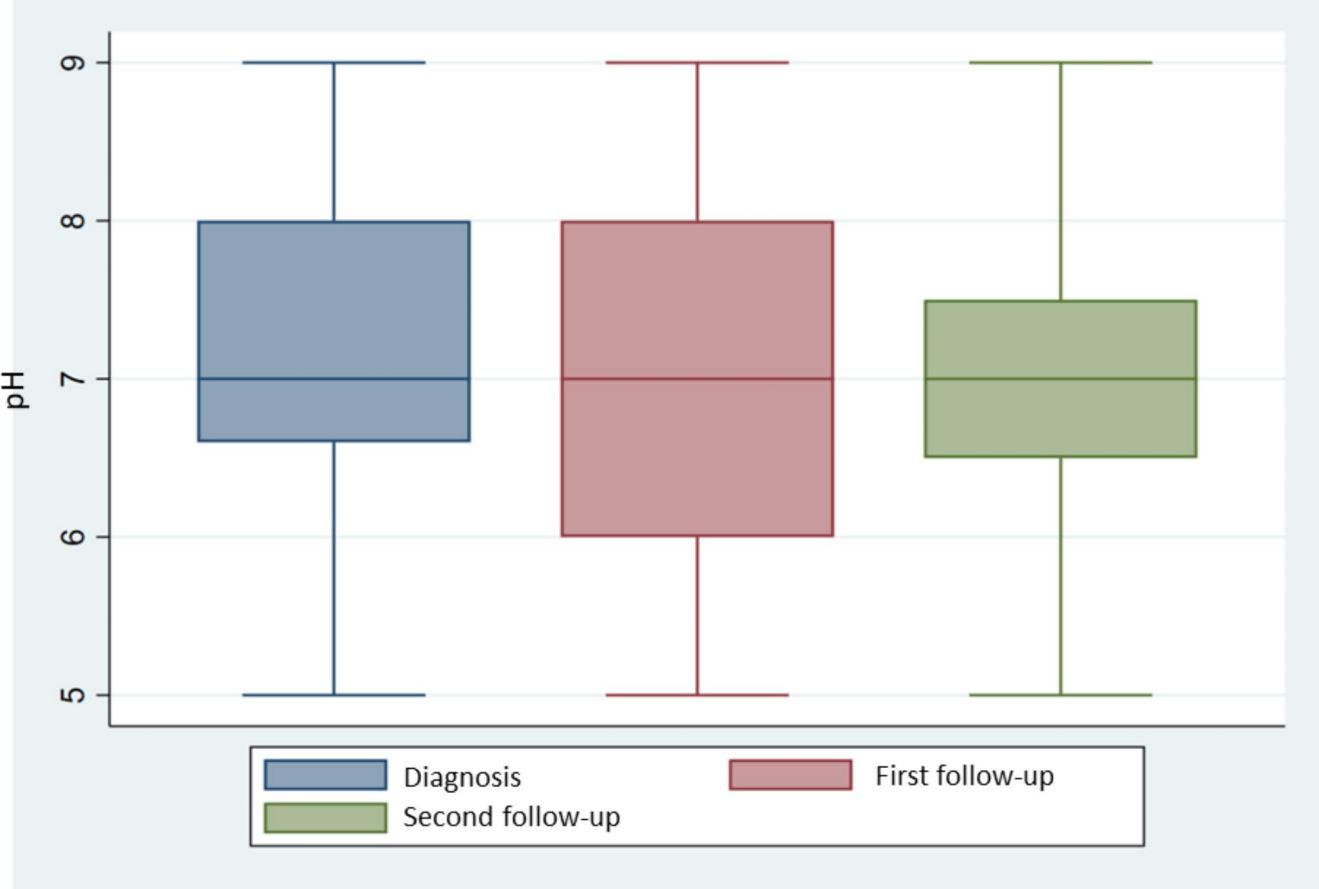
Box and whisker plots of pH in urine measured at diagnosis (blue), at first follow-up (red) and second follow-up (green) for respectively 22, 22 and 21 dogs. Only dogs with available data for all three datapoints are included in the figure

### Characteristics for dogs with cystine urolithiasis

#### Diet at the time of cystine diagnosis

Data on feeding habits at the time of diagnosis according to owners were available for 79% (n = 67/85) of the patients with cystine urolithiasis. 28.4% (19/67) of the dogs were on a raw diet at the time of diagnosis. Almost half of the dogs (47.8%) were fed a type of commercial dry food according to the owners. 23.9% (16/67) reported that their dog was fed a mixed diet at the time of diagnosis. Components of the different mixed diets varied a lot. Some reported a mix of two different commercial brands, others reported to be feeding table-scraps together with a commercial dry, raw diet or both. Exact information on the different components and relative amounts of these in the mixed diets were not available. After surgical treatment for cystine uroliths, 60% (n = 51/85) were fed a commercial diet as a preventive measure, of which 80% (n = 41/51) of the patients were fed Hill´s u/d and 16% (n = 8/51) were fed Royal Canin Urinary U/C low purine. Two dogs (n = 2/51) were fed Royal Canine Urinary SO after surgery. Information about diet after the diagnosis was unavailable for 40% (n = 34/85).

### Treatment

Almost all cystine cases (90.6% (n = 77/85)) were treated surgically at the time of diagnosis to remove cystine uroliths. Seven of these patients had two surgeries and two of them had three surgeries due to recurrence. It was not known if the recurrence was clinical or detected by incidence. Four patients (4.7% (n = 4/85)) were treated with voiding urohydropropulsion to remove stones and four (4.7% (n = 4/85)) were not treated at all. Eight of the dogs were treated with tiopronin to prevent recurrence.

### Urine analysis

Nearly all patients with cystine urolithiasis (95%, (n = 81/85)) had results from urine analysis available at the time of diagnosis; the mean pH was 7.1 (SD = 0.8, n = 73) and mean specific gravity was 1.036 (SD = 0.01, n = 69). The data was normally distributed as indicated the Shapiro-Wilk test of normality. After feeding the recommended diets, no increase in urine pH or lowering of urine specific gravity on the follow-up visits were evident (Table 3 and Fig. 3). Twelve dogs lacked data on urine pH at surgery, and all together 43 dogs had available pH at time of surgery and first control.

**Table 3:** Urine analysis at diagnosis and on follow-up visits for cystine cases

Follow-up visit	0* (n)	1 (n)	2 (n)	3 (n)	4 (n)	5 (n)	6 (n)
pH (mean ± SD)	7.1 ± 0.8 (73)	6.9 ± 1.1 (47)	6.9 ± 1.1 (29)	7.05 ± 1.3 (20)	6.6 ± 1.2 (11)	6.3 ± 0.8 (10)	5.8 ± 0.8 (9)
Urine SG (mean ± SD)	1.036 ± 0.01 (69)	1.034 ± 0.01 (46)	1.031 ± 0.01 (29)	1.033 ± 0.01 (19)	1.035 ± 0.01 (10)	1.036 ± 0.02 (5)	1.032 ± 0.01 (5)

* 0 is time of diagnosis

** number of observations in parenthesis.

SG: Specific gravity.

### COLA analysis

COLA analysis was performed for 39 patients. Two patients were excluded from the group as mentioned in the methods section. Half of the patients (n = 19/37) had only one COLA analysis done, while the remaining half performed two or more COLA analysis 49% (18/37).

Most of the patients (n = 30/37) had the first COLA analysis done before neutering and around the time of diagnosis. Of these patients, 93% (n = 28/30) had elevated cystine values at this time and 7% (n = 2/30) had normal cystine values. Both cystine and total sum of COLA were significantly reduced after neutering by castration or by injection of deslorelinacetat implant (P < 0.0001) (Table 4).

When the first COLA analysis was performed 65% (n = 24/37) of the patients had already started a preventive diet such as Hill´s u/d or RC low purine. 83% (n = 15/18) of the patients were fed one of the recommended preventive diets when the second COLA analysis was performed.

**Table 4:** Summarize statistics of COLA analysis prior to and after neutering. Only dogs (n = 15) with urine COLA values available before and after neutering were included

	Cystine Umol/g creatinine	Ornithine umol/g creatinine	Lysine umol/g creatinine	Arginine umol/g creatinine
Mean value at diagnosis	743.5 ± 619.6	107.4 ± 109.2	1188.5 ± 1576.4	150.6 ± 93.0
Mean value after neutering	151.3 ± 182.5	44.4 ± 67.8	315.0 ± 315.0	34.9 ± 41.9

## Discussion

### Increased occurrence of cystine uroliths in Norway from 2015 to 2020

In this study cystine uroliths comprised 24.6% of the total number of urolith cases in the clinics between 2015 and 2020. We found a significant increase in the diagnosis of cystine uroliths during these five years, with a peak in 2019. Our findings concur with earlier reports from Norway and abroad describing an increase in occurrence of cystine uroliths [6–8].

### Breed as a risk factor for cystine urolith formation

The breeds that were most commonly affected with cystine urolithiasis in this study were Staffordshire bull terriers, Danish Swedish farmdogs, Chihuahuas, Shetland sheepdogs and French bulldogs in descending order. These breeds are also frequently seen in previous studies investigating cystine urolithiasis [9–11]. Other breeds with clinically relevant higher risk of developing cystine-containing uroliths are Mastiff, Australian cattle dog, Pitbull terrier, French bulldog, Rottweiler and English bulldog. Type III cystinuria is suggested to occur in Staffordshire bull terriers as well as in several other breeds [10]. For Staffordshire bull terrier and Chihuahua among others, a genetic basis has also been suggested because of one or both of the two following criteria: increased risk of cystine urolith formation based on data from urolith analyzing laboratory, or multiple related cystinuric dogs or test mating that produced cystinuric dogs [3, 7].

The higher occurrence of cystine uroliths among Staffordshire bull terrier and Chihuahua could be influenced by the fact that Staffordshire bull terrier and Chihuahua have been among the five most popular breeds in Norway the last ten years (NKK, Norwegian Kennel Club. 2019). Still the popularity of these breeds could not fully explain why the number of cystine uroliths is increasing, as both registration numbers for Chihuahua and Staffordshire bullterrier have been quite stable for the last ten years (NKK).

### Age in dogs diagnosed with cystine uroliths

Patients with cystine uroliths where significantly younger compared to patients with calcium containing uroliths [7]. This has also been reported previously and may be explained by the genetic background of cystinuria, which may lead to an early development of cystine uroliths [9].

The age at diagnosis varied a lot from one to 12 years old. This could be explained by the fact that patients with genetic mutations seem to develop cystine uroliths earlier than the ones with androgen dependent cystinuria [12]. Our material probably included both dogs with androgen dependent and “genetic” cystinuria. This may explain the variation of age at diagnosis in dogs with cystine uroliths.

### Gender/neuter status as a risk factor for cystine urolith formation

All of the dogs diagnosed with cystine uroliths were intact males. This was in contrary to dogs with calcium oxalate, magnesium-ammonium-phosphate and unknown uroliths, where the groups represented both neutered and intact males and females.

Also, in other studies there has been an association between neuter status and the risk of developing cystine uroliths, with increased risk in entire males [10]. The underlying cause of the androgen-dependent (Type III) cystinuria is still unknown [10].

Earlier studies describe a decrease in the relative frequency of cystine uroliths over the years [9]. The reason for a marked drop in relative frequency of cystine uroliths in Germany from 1979 to 2013 was not clear but the increasing routine neutering was considered a possible explanation.

In Norway, the Law of Animal Welfare does not permit routine neutering of dogs, and so the proportion of entire males is likely to be larger in Norway compared to many other countries. Still, since the law has been applicable for a long time, it is unlikely that this would lead to the increase in cystine urolithiasis seen in this study.

### Diet as a risk factor for cystine urolith formation

Almost half (32/68) of the clients reported to feed a commercial dry food diet. Many of the dry food brands mentioned by the owners could be classified as high protein diets (> 30% crude protein).

Diets that promote formation of acidic concentrated urine are considered risk factors for cystine urolithiasis in susceptible dogs. These include high-protein dry diets, especially those rich in methionine. Dietary methionine is a sulfur-containing amino acid that is a precursor for cystine. Methionine is common in many animal-derived nutrients and some plant-derived nutrients [4]. Besides most meat, other foods high in methionine include eggs, wheat and peanuts [13].

Due to the retrospective nature of this study, there is uncertainty about exactly what type of food owners gave and to what extent they gave it. Some owners (19/68) reported to be feeding a commercial raw food diet. This type of diet is not considered to have a high protein content (< 30% crude protein), but is probably rich in methionine.

To identify diet as a risk factor for cystine urolithiasis further investigations need to be done.

### COLA analysis

Most of the dogs in our study had an increased level of cystine and an increased total of cystine, ornithine, lysine and arginine on their first COLA-analysis.

In a study investigating urinary excretion of amino acids in normal and cystinuric dogs, there was a significant correlation between the level of cystine and the level of ornithine, lysine and arginine [11]. The fact that dogs with increased values of cystine also had increased values of ornithine, lysine and arginine, suggests that there is a common reabsorption mechanism, as it is in humans. This is why all of the four amino acids may show increased values in cystinuric dogs.

A previous study reported that the excretion of cystine in the urine of cystinuric dogs is variable [11], and that there may be an overlap between normal dogs and dogs with cystine uroliths. The level of cystine in urolith forming dogs varied from normal to approximately 100 times normal. There may be variations in cystine levels during the day [1]. Not all dogs with cystinuria develop uroliths, and other factors than excretion of cystine must be considered as causes of cystine urolith formation [11]. This may explain why a few of the dogs in our study had normal cystine and total COLA levels at diagnosis.

There was a significant decrease in both cystine and total COLA levels

after neutering and change of diet in our study. This could support the fact that neutering will prevent cystine urolithiasis in many cases.

Because of the retrospective nature of this study, there was no standardized timing for the follow-up COLA-analysis after neutering or diet change. Some of the dogs had already been fed a low-protein diet for some days when the first COLA-analysis was performed. As changing the diet to a decreased protein, urine alkalizing canned food will decrease the urine cystine excretion with 20–25% in 24 h compared with a canned maintenance diet, the cystine values in these dogs may have been even higher than before the diet change. [4]

The COLA-values will decrease with the age of the dog [1]. This could be because of the fact that older dogs are more likely to be neutered, or that the level of testosterone decreases with age [14].

### Treatment

In our material almost all of the dogs went through surgery as treatment for cystine urolithiasis. This despite that the ACVIM consensus suggests to consider medical dissolution of cystine uroliths as first-line treatment if the uroliths can be bathed effectively in urine and is not causing obstruction [4]. A possible explanation for the high number of surgically treated patients could be that several of the dogs presented with urinary obstruction at the time of diagnosis. Few of the patients in this study were treated with voiding urohydropropulsion. We think this number will increase in the future. The importance of regular follow-ups with ultrasound of the urinary bladder and urohydropropulsion of cystine uroliths in an early stage has been acknowledged more recently.

### Thiol-containing Drugs

Drugs that increase the solubility of cystine in urine contain a thiol group that can dissociate and then bind with the sulfide moiety of cysteine. The resulting complexes are more soluble in urine than cystine (dicysteine). 2-MPG (tiopronin) is a second-generation cystine-chelating agent. It decreases the concentration of cystine by thiol-disulfide exchange reaction [13].

Studies in humans and dogs indicate that the drug is highly effective in reducing urinary cystine concentration and has less toxicity than D- Penicillamine [1]. Most dogs tolerate the treatment with tiopronin well. Mild to moderate proteinuria, aggressiveness, myopathy, vesicular skin disease, thrombocytopenia, anemia and elevated hepatic enzymes have been reported. All side effects disappeared when treatment was stopped [1, 11].

Only eight of the dogs in this study were treated with tiopronin (40 mg/kg) to prevent recurrence of cystine uroliths. Tiopronin is not registered for use in animals in Norway. It has been difficult to get hold of and is quite expensive. This could play a role in why it has not been used more often although it is recommended [4].

### Preventive measures for cystine uroliths

Because cystinuria is as an inherited defect, urolith recurrence is common unless prophylactic therapy has been initiated. Current recommendations for dissolution and prevention of cystine uroliths include a reduction in the urine concentration of cystine and an increase in the solubility of cystine in the urine. This can be accomplished by various combinations of dietary modification, diuresis, administration of thiol-containing drugs, and alkalinization of urine [13]. Increasing urine volume to reduce cystine concentration is likely to be of substantial benefit. Feeding moist rather than dry food is recommended. If feeding a dry diet adding water should be done. A specific gravity value of less than 1.020 should be attained if possible [13].

The clients in this study were educated about different preventive measures for recurrence of cystine urolithiasis at the time of diagnosis, and 58% (49/85) of the patients were fed a preventive diet after the time of diagnosis.

The solubility of cystine is pH dependent. Cystine is relatively insoluble in acidic urine but becomes more soluble in alkaline urine [15]. In dogs, cystine is twice as soluble at a urine pH of 7.8 compared to a urine pH of 5 [16]. Potassium citrate could be used to sustain a urine pH of approximately 7.5 [13]. Adding potassium citrate seems to be a challenge for the clients because of the large volume that needs to be given orally to increase the urine pH. This is probably why only a few of these dogs were treated with potassium citrate.

In our material there was no significant increase in urine pH and no general lowering of the specific gravity in the follow up urine samples. This was despite the veterinarian trying to motivate the owners to preventive measures like increasing water intake and feeding a preventive moist diet. Some of the commercial cystine preventing diets are also added potassium citrate and calcium carbonate to help alkalinize urine. Most of the clients changed their dogs’ diet shortly after surgery and urolith analysis.

One reason for the lack of increase in pH of the urine despite owners feeding a preventive diet, could be that the urine samples were collected at the morning when the dog has been fasted. Due to gastric acid secretion a post-prandial urine sample is the most alkaline [17]. Sampling urine 4–6 h after the dog’s meal could give a more accurate measure for increase in urine pH after changing to a low-protein diet and/or adding potassium citrate.

The lack of reduce in urine specific gravity could also be explained by the use of morning urine samples. Urine is suspected to be at it most concentrated in the morning.

Because this was a retrospective study, there were no conformity regarding when the urine samples were retrieved in relation to time of the day or time after feeding. This may have affected the pH and specific gravity of the urine, and a more standardized routine for urine sampling may have given a different result.

## Conclusions

There was a significant increase in the prevalence of cystine uroliths in Norway from 2015 to 2020. There was a younger mean age for occurrence of cystine urolithiasis in contrary to calcium oxalate. An overrepresentation of entire males when it comes to cystine urolithiasis was found. Both cystine and total sum of COLA reduced significantly after neutering by castration or by injection of deslorelinacetat implant. The breeds with increased occurrence of cystine uroliths was Staffordshire bull terrier, Chihuahua and Danish Swedish farmdog. Changing the diet to what is generally recommended for cystine urolithiasis did not influence pH and urine specific gravity.

## Data Availability

The datasets used and/or analyzed during the current study are available from the corresponding author on reasonable request.

## Declarations

### Prior publication

Data have not been published previously.
